# Supplementary material for: Patterns of Intron Gain and Loss in Fungi
Source: PLoS Biol. 2004 Nov 30;2(12):e422. doi: 10.1371/journal.pbio.0020422 (PMC532390; doi:10.1371/journal.pbio.0020422)
Supplement: Table S1 — Also available at http://genes.mit.edu/NielsenEtAl/. (4.3 MB ZIP). [file pbio.0020422.st001.zip › NielsenEtAl/html/530.html]

AN4992.1.NCU02416.1.MG03560.1.FG06375.1


```
 CLUSTAL W (1.82) Multiple Sequence Alignments - Introns Inserted


Sequence 1: MG03560.1	638 aa
Sequence 2: FG06375.1	630 aa
Sequence 3: NCU02416.1	634 aa
Sequence 4: AN4992.1	623 aa
Alignment Length: 646 aa
Number Identitical Residues: 324 aa
Alignment Score (without introns) 16319


MG03560.1 	MATALRRRPVVHE---ESPSPTPTPTDTPRTASPAREEK--EKIHVVHHK-PRTRKRRNT
NCU02416.1	MATTVRRRLVGKDIECEIASETASETATSREPSPDNQQREVTSIFKAIHKRSKTRKRKTT
FG06375.1 	MGKSTLRRRNGQD---ATNNDSADADDTPREESPTAEPT--THVRVVQHAVPRTRKRRNT
AN4992.1  	----MIRRRQAKDNSDDVQAPAPADSSPEKQLPPAVAKP--KNERKQSFITKPKSKRRNG
          	      **   :: .      :.    . :  .*              .    . **:. 

MG03560.1 	FIFLLGSLSGIIAAG-FAKTNDLIDFPEIGDLGMSMDSLLDVLPAGLVKDMSDLV0RGER
NCU02416.1	AIFLLGSLFGIIAAGFFAKSNDLIDLPLPELKELSVDSLFEVLPAGFVKEMRDLM0NGER
FG06375.1 	FVFFLGSLFGIIAAGFFASSNDLIDLPEFTDL--SMDNLMDVLPAGLIKDMRDLV~QGER
AN4992.1  	LIFLLGGVFGIFCAVFFAQQQDVISLDSLMDLN--IDSLMDVIPQSIMRDAREFS0QHER
          	 :*:**.: **:.*  **. :*:*.:         :*.*::*:* .::::  ::  . **

MG03560.1 	DFVDSSSEPFSVGVKAKTEGLQAHFPVVMVPGVISTGLESWGTANSSRPYFRKRLWGSWT
NCU02416.1	DLIES-DDAFSVGMKMRAEGLSADHPIVMIPGVISTGLESWSTSELSLPYFRKRLWGSWT
FG06375.1 	DIAES-YEPFSVGEKARSEGLGVHHPMIMIPGVISTGLESWGTANISKPYFRKRLWGSWT
AN4992.1  	DTVSY--DAFSVGLHLRSQGVEAKHPIIMIPGVISTGLESWGTSPTSLMYFRRRLWGSWS
          	*  .   :.**** : :::*: ...*::*:***********.*:  *  ***:******:

MG03560.1 	MMRALVLDKEAWKRHVMLDKVTGLDPPGIKLRAAQGFDATDFFITGYWIWNKILENLAAL
NCU02416.1	MMRALVMDKEAWKRHIMLDKTTGLDPPGIKLRAAQGFDATDFFITGYWIWNKILENLASL
FG06375.1 	MMRALVMDKEVWKKHVMLDKRTGLDPPDVKLRAAQGFDATDFFITGYWIWSKIFENLASI
AN4992.1  	MMRALVLDKTEWKNHIMLDKETGLDPPGIKLRAAQGFDATDFFITGYWIWNKILENLASI
          	******:**  **.*:**** ******.:*********************.**:****::

MG03560.1 	GYDTNTAFTAAYDWRLSYPNLEVRDQYFTRLKMAIETATITSGRKAVLVSHSMGSQVLFY
NCU02416.1	GYDPTNSYTAAYDWRLAYANLEVRDHYFSRLKSYIEQAVFIHKKKVVLTSHSMGSQVLFY
FG06375.1 	GYDPTNSFTAAYDWRLSYPNLEVRDRYFTRLKSHIEIAVATEDKKVVLASHSMGSQVLYY
AN4992.1  	GYDPTNAYTAAYDWRLSYLNLEARDHYFSRLKSYIETAVQVRGEKVTLASHSMGSQVVLF
          	***...::********:* ***.**:**:***  ** *.    .*..*.********: :

MG03560.1 	FFHWVASQKGGKGGDDWVDRHVDSWINISGCMLGAVKDLTAVLSGEMRDTAQLNAFAVYG
NCU02416.1	FFHWVASKKGGQGGQDWVERHVDSWINISGCMLGAVKDVTAILSGEMRDTAQMNAFAVYG
FG06375.1 	FLHWVQSERGGRGGPDWVERHIDAWINISGCMLGAVKDLTAVLSGEMRDTAQLNPFAIYG
AN4992.1  	FFKWVENPAHGKGGSDWVNRHIANWINISGCMLGAAKGLTAVLSGETRDTALLNSFAVYG
          	*::** .   *:** ***:**:  ***********.*.:**:**** **** :*.**:**

MG03560.1 	LEKFLSKEERAQMFRHMPGISSMLPLGGEAVWGNTTWAPDDLPGQQKSYGPVLNFRKGYG
NCU02416.1	LEKFLSKEERAEIFRAMPGISSMLPMGGNAVWGELDWAPDDQPGQGHSYGSLLNFRAGQN
FG06375.1 	LEKFLSKEERAEIFRGMPGISSMLPIGGNSVWGNLTWAPDDLPGQNRSYGSLLNFRVGS-
AN4992.1  	LEKFLSREERAEIFRAMPGISSMLPKGGEAVWGNSTWAPDDQPGQKITYGNILNFRETN-
          	******:****::** ********* **::***:  ***** ***  :** :****    

MG03560.1 	YNKTAPTRNMTVSTAMQYLFDTSEDWYQNNVKRSYSHGVAHTKAEVDANEDDPTKWINPL
NCU02416.1	-VSTTPDRNFTIEDAMDYILDTTDDWYRDQVKGSYSHGVAKTRAEVEANENDPRKWINPL
FG06375.1 	-NWTTPDRNFTVEEGVSYLLNTTEDWYQDQIKGSYSRGIAHSIDEVEANENDPKKWINPL
AN4992.1  	--STFTQKNLTVPESLDYLLDQSEPWYRDQVLGSYSHGVAHTTAEVEANENDPRTWLNPL
          	   * . :*:*:  .:.*::: :: **::::  ***:*:*::  **:***:** .*:***

MG03560.1 	ETRLPLAPNLKIYCFYGVGKPTERAYYYRTPEFPALTNLSITIDTGLTRGE-IDHGVVLG
NCU02416.1	ETRLPLAPSLKVYCFYGVGKPTERAYFYRAPDPGTTTHLKMTIDTTLTQGH-IDHGVILG
FG06375.1 	ETRLPLAPSLKIYCFYGVGKPTERGYFYKPPDQPSLTNLNITIDTGYTEGD-VDHGVVMG
AN4992.1  	EARLPLAPDMKLYCFYGVGKPTERSYFYQE-ERDPLVNLNVSIDTTVTTADGTDHGVVLG
          	*:******.:*:************.*:*:  :  . .:*.::***  * ... ****::*

MG03560.1 	EGDGTVNLISSGYMCNHGWKYKRYNPAGSKVTVVEMPHEPDRFNPRGGPNTA~DHVDILG
NCU02416.1	EGDGTVNLMSLGYMCNKGWKMKRYNPAGSKITVVEMPHEPERFNPRGGPNTA~DHVDILG
FG06375.1 	EGDGTVNLLSTGYMCNHGWNMKRYNPAGVKVTVVEMPHEPDRFNPRGGPRTA~DHVDILG
AN4992.1  	EGDGTVNLLSTGYMCAKGWHIKRYNPSGIKIKVYEMPHEPDRFSPRGGPNTG1DHVDILG
          	********:* **** :**: *****:* *:.* ******:**.*****.*. *******

MG03560.1 	RQTLNELILQVAAGKGESIQNHLVSNIMKYAAKVKVYDDYPQQGPQPA
NCU02416.1	RQNLNEYILKVAAGRGDTIEDFITSNILKYVEKVEIYEE---------
FG06375.1 	RYNLNELLLRVASGKGDTITNYVVSNIKEYASRVKIYDDEETS-----
AN4992.1  	RASLNDLILRVAGGRGDEIEDTFVSRIKEYAERVKIYEE---------
          	* .**: :*:**.*:*: * : ..*.* :*. :*::*::
```
